# Prokaryotic Expression, Purification and Immunogenicity in Rabbits of the Small Antigen of Hepatitis Delta Virus

**DOI:** 10.3390/ijms17101721

**Published:** 2016-10-20

**Authors:** Vera L. Tunitskaya, Olesja V. Eliseeva, Vladimir T. Valuev-Elliston, Daria A. Tyurina, Natalia F. Zakirova, Olga A. Khomich, Martins Kalis, Oleg E. Latyshev, Elizaveta S. Starodubova, Olga N. Ivanova, Sergey N. Kochetkov, Maria G. Isaguliants, Alexander V. Ivanov

**Affiliations:** 1 Engelhardt Institute of Molecular Biology, Russian Academy of Sciences, Vavilov str. 32, Moscow 119991, Russia; ve_tun@mail.ru (V.L.T.); gansfaust@mail.ru (V.T.V.-E.); tyurina.dascha@gmail.com (D.A.T.); nat_zakirova@mail.ru (N.F.Z.); o.a.khomich@gmail.com (O.A.K.); estarodubova@yandex.ru (E.S.S.); olgaum@yandex.ru; (O.N.I.); kochet@eimb.ru (S.N.K.); 2 Gamaleya Research Center of Epidemiology and Microbiology, Gamaleja str. 16, Moscow 123098, Russia; oleglat80@mail.ru (O.V.E.); olesenka80@mail.ru (O.E.L.); 3 A Kirchenstein Institute of Microbiology and Virology, Research Department, Riga Stradins University, Dzirciema iela 16, Riga LV-1007, Latvia; martins.kalis@rsu.lv

**Keywords:** hepatitis delta virus, prokaryotic expression, protein purification, rabbit immunization

## Abstract

Hepatitis delta virus (HDV) is a viroid-like blood-borne human pathogen that accompanies hepatitis B virus infection in 5% patients. HDV has been studied for four decades; however, the knowledge on its life-cycle and pathogenesis is still sparse. The studies are hampered by the absence of the commercially-available HDV-specific antibodies. Here, we describe a set of reproducible methods for the expression in *E. coli* of His-tagged small antigen of HDV (S-HDAg), its purification, and production of polyclonal anti-S-HDAg antibodies in rabbits. S-HDAg was cloned into a commercial vector guiding expression of the recombinant proteins with the C-terminal His-tag. We optimized S-HDAg protein purification procedure circumventing a low affinity of the His-tagged S-HDAg to the Ni-nitrilotriacetyl agarose (Ni-NTA-agarose) resin. Optimization allowed us to obtain S-HDAg with >90% purity. S-HDAg was used to immunize Shinchilla grey rabbits which received 80 μg of S-HDAg in two subcutaneous primes in the complete, followed by four 40 μg boosts in incomplete Freunds adjuvant. Rabbits were bled two weeks post each boost. Antibody titers determined by indirect ELISA exceeded 10^7^. Anti-S-HDAg antibodies detected the antigen on Western blots in the amounts of up-to 100 pg. They were also successfully used to characterize the expression of S-HDAg in the eukaryotic cells by immunofluorescent staining/confocal microscopy.

## 1. Introduction

Hepatitis delta virus (HDV) is a defective viroid-like agent which infects patients on the background of a newly acquired or an established infection with hepatitis B virus (HBV) (co-, and super-infection, respectively), in both cases aggravating liver disease. Co-infection with HDV and HBV in 95%–98% cases resolves as acute hepatitis B, but can also cause a severe fulminant hepatitis. The latter results in a massive necrosis of hepatocytes, liver failure, and death in up to 80% of patients, if they cannot undergo liver transplantation. Superinfection, in contrast, results in the chronic disease in the majority (80%–90%) of cases. Chronic HBV/HDV infection presents a more severe liver disease than chronic HBV mono-infection and is manifested by an accelerated fibrosis progression, early decompensation in the settings of established cirrhosis, and an increased risk of hepatocellular carcinoma attributed to the rapid development of cirrhosis (for a review see [1]). Treatment of chronic HDV infection is difficult as it does not have an enzymatic function to target [2]. The only established therapy is treatment with the pegylated-interferon α, effective in 25%–30% of cases [3,4]. Due to the introduction of massive HBV immunization of the newborn babies, and gradual extension of the HBV vaccination to older subjects, risks of acquisition of HDV infection have notably decreased [5]. However, infection with HDV is still a major health problem affecting 15 to 20 million people worldwide, specifically in the countries where HBV vaccination is not performed, and in the regions where HDV infection is endemic, as in the Middle East, Mediterranean area, Amazonian region, some African countries, and parts of the Russian Federation [6,7].

HDV genome is presented by circular antigenomic RNA. It is replicated by the host RNA polymerases generating genomic and mRNA forms. HDV RNA contains a single open reading frame (ORF) encoding a protein of 195 amino acid residues referred to as the small HDV antigen (24 kDa; S-HDAg). Antigenomic HDV RNA is partially edited by dsRNA-adenosine deaminase 1 (ADAR) [8] that converts the UAG stop codon to an amber UIG codon. The latter results in the elongation of ORF which generates an extended 214 amino acid long protein referred to as the large HDV antigen (27 kDa; L-HDAg). None of them exhibits any enzymatic activity. In the virus life cycle, S-HDAg and L-HDAg act as the regulatory proteins. S-HDAg is important for virus replication, whereas L-HDAg inhibits replication and leads to the assembly of the virion [9,10]. During virion assembly, S-HDAg and L-HDAg form a capsid for antigenomic RNA. At the later stages of the virion production, the capsids get surrounded by HBV surface antigens [10,11].

Patients with acute self-limiting HBV-HDV coinfection exhibit a panel of HDV specific serological responses. Serum HDV RNA and HDV antigens may be detected early, concurrently with the detection of HBV surface antigen (HBsAg). Disappearance of HDV antigen is followed by the seroconversion to anti-hepatitis D antibodies, first IgM, and then IgG. HDV superinfection of HBV carriers is manifested by the appearance of HDAg and HDV RNA, with a simultaneous reduction of HBV replication. Patients with chronic HDV infection maintain high titers of anti-HDV IgM and IgG [12]. Diagnosis of HDV infection based on serological testing and confirmation of replication by nucleic acid testing [13]. Several in-house assays and commercial PCR have been developed but their capacity to detect all HDV genotypes remains questionable [14]. However, these assays are not yet standardized and the results from different laboratories are often not comparable [15,16]. Major differences in the sensitivity were also found for the immune assays detecting HDAg [16,17]. Moreover, there are no commercially available antibodies for the detection of HDV antigen even for the research purposes. Anti-HDAg antibodies were raised in rabbits [18,19], however, their availability for the scientific community was always limited. Our goal was to fill this gap and generate antibodies against HDV antigens that could be used in a variety of HDV-specific immune assays. This requested expression of the small HDV antigen in quantities sufficient for raising polyclonal antibodies in laboratory animals, such as rabbits. Hereby, we describe the detailed protocols for the expression and purification of the small antigen of HDV in *Escherichia coli*, generation of polyclonal anti-HDV rabbit antibodies. The level of antibodies directed against S-HDAg in rabbit serum was assessed by ELISA, and antibody affinity tested by Western blot and immunofluorescence/confocal microscopy.

## 2. Results

### 2.1. Expression and Purification of Small HDV Antigen

**Figure 1:**
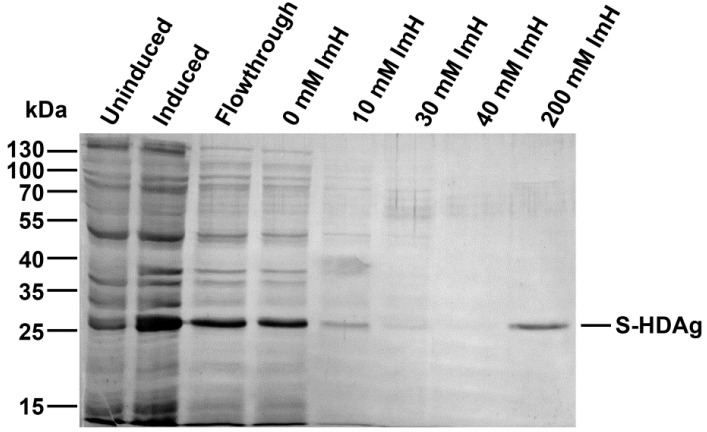
Expression and purification of the His-tagged small antigen of hepatitis delta virus (S-HDAg). SDS-PAGE analysis of the lysates of uninduced and induced *E. coli* Rosetta (DE3) cells, and of the fractions obtained during purification of the recombinant antigen on the Ni-nitrilotriacetyl agarose (Ni-NTA-agarose) column. Concentration of imidazole used for elution of His-tagged S-HDAg from the Ni-NTA-agarose column is depicted over the wells. Molecular mass markers, in kDa, are given to the left.

Raising of specific antibodies requires sufficient amounts of pure antigen, in this case S-HDAg. A plasmid for the expression of S-HDAg, pET-21d-SHDAg, was constructed based on the widely used pET-21d vector for bacterial expression of the C-terminally His-tagged recombinant proteins. S-HDAg was expressed in the Rosetta (DE3) *E. coli* strain which carries a plasmid encoding “rare” tRNAs required for the expression of the mammalian and viral genes. Plasmid pET-21d-SHDAg transformed into the Rosetta (DE3) strain directed efficient production of S-HDAg (Figure 1).

The protein was purified using Ni-nitrilotriacetyl agarose (Ni-NTA-agarose) column. Earlier, Ding et al. described low affinity of S-HDAg to the Ni-NTA-agarose [20]. Indeed, during the isolation procedure we observed that despite very high protein levels in the cell lysate, only a minor fraction of S-HDAg (<10%) was bound to the column (Figure 1). This may be due to the globular conformation adopted by the major portion of the protein, preventing the hexahistidine tag from binding to the resin. This problem was partially resolved by using large volumes of cell culture (1–2 L) and recycled loading of the cell lysate onto the resin (lysates were allowed to pass the column two to three times instead of one). This approach typically provided 1 mg of S-HDAg per 1 L of *E. coli* culture. Antigen purification also required large (>10 resin volumes) volumes of the wash buffers. Once obtained, the protein was dissolved in a Tris-HCl buffer containing 10% glycerol, 300 mM NaCl, and 1 mM 2-mercaptoethanol and stored at +4 °C. In these conditions protein was intact for at least 50 h, longer incubations led to gradual protein degradation. For a longer-term storage, S-HDAg was dialyzed against the same buffer supplemented with 50% glycerol, aliquoted and kept frozen at −20 °C.

### 2.2. Rabbit Immunization and Evaluation of Rabbit Anti-S-HDAg

**Figure 2:**
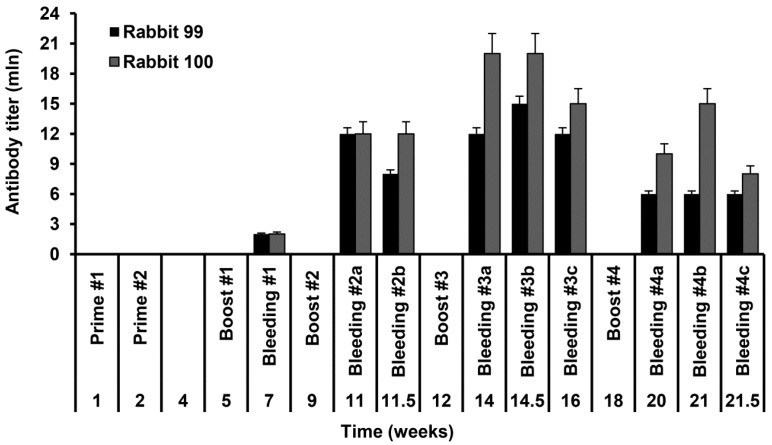
Level of anti-S-HDAg antibodies in the sera of Shinchilla grey rabbits immunized with the His-tagged S-HDAg. Data is presented as the end-point antibody titer. *X*-axis depicts time points of immunizations and bleedings in weeks.

Due to instability of S-HDAg, all immunizations were performed within the first 24 h after protein purification. Ten-week-old female rabbits were primed with subcutaneous and repeatedly boosted with intravenous injections of the freshly purified S-HDAg. Each animal received the total of 240 µg of the protein. Sera collected two weeks after each boost were assessed for the presence of anti-S HDAg antibodies by ELISA on plates coated with a freshly prepared S-HDAg lot. The initial antibody titer after the first boost equaled to 2 × 10^6^, and the highest antibody titer after the third boost reached 2 × 10^7^ (Figure 2). Further boosting led to a decrease of antibody titer as detected by ELISA (Figure 2).

### 2.3. Application of Anti-S-HDAg Antibodies for Western Blotting

**Figure 3:**
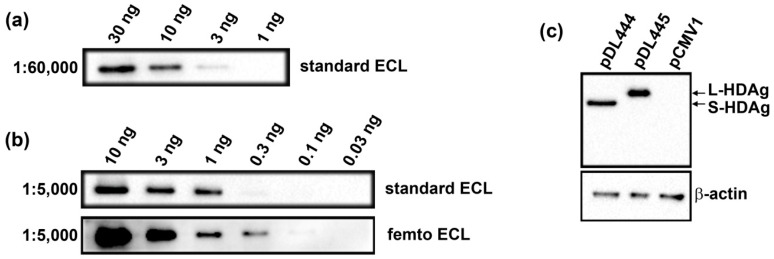
Detection of hepatitis delta virus (HDV) antigens using Western blotting with polyclonal rabbit sera specific to S-HDAg. (**a**,**b**) Detection of the recombinant S-HDAg in the amounts of 30 to 0.3 ng using rabbit serum #100-3a diluted as 1:60,000 (**a**) or 1:5000 (**b**) by the standard ECL, or sensitive femto ECL reagents (S-HDAg amounts are shown above the lanes in panels **a** and **b**); (**c**) Expression of small and large HDV antigens in Huh7.5 cells. In brief, Huh7.5 cells were transfected with pDL444 or pDL445, respectively, and control cells, with empty vector pCMV1 (plasmid used is depicted on top of each lane) and grown for additional 48 h. Blots were first stained with anti-S-HDAg serum diluted at 1:5000 (**c**, upper panel), then stripped and re-stained with mouse monoclonal against actin (**c**, lower panel).

The next step was to evaluate if polyclonal anti-S-HDAg sera can detect HDV antigens by Western blotting. First, we tested the reactivity of rabbit sera with the recombinant S-HDAg on the example of sera from rabbit no. 100 (bleeding 3a; #100-3a). Serum #100-3a diluted 1:5000 to 1:20,000 applied in Western blotting with the standard Pierce ECL Western Blotting Substrate readily detected up to 1 ng, and sera diluted 1:60,000, 3 ng of the antigen (Figure 3a,b, respectively). Western blotting with enhanced chemiluminescence (ECL) reagents of an enhanced sensitivity (such as Super Signal West Femto Maximum Sensitivity Substrate) detected as low as 100–300 pg of S-HDAg (Figure 3b). Sera of rabbit no. 99 (bleeding 3b; #99-3b) demonstrated similar sensitivity (see Supplementary Figure S1). Further, we tested if hyperimmune anti-S-HDAg sera (hereby, #100-3a) detects small and large HDV antigens expressed in the hepatocytes. For this, Huh7.5 cells were transfected with plasmids directing eukaryotic expression of S-HDAg and L-HDAg (pDL444 and pDL445, respectively, see Methods). Empty pCMV1 vector was used as a negative control. Lysates of the transfected Huh7.5 cells were resolved by SDS-PAGE and analyzed by the Western blotting (Figure 3c). Both antigens were readily detected already 30 h posttransfection, their amounts increased notably after 48 h. Notably, Western blotting did not reveal any unspecific bands (Figure 3c and Figure S2). 

### 2.4. Application of Anti-S-HDAg Antibodies for Immunofluorescence

**Figure 4:**
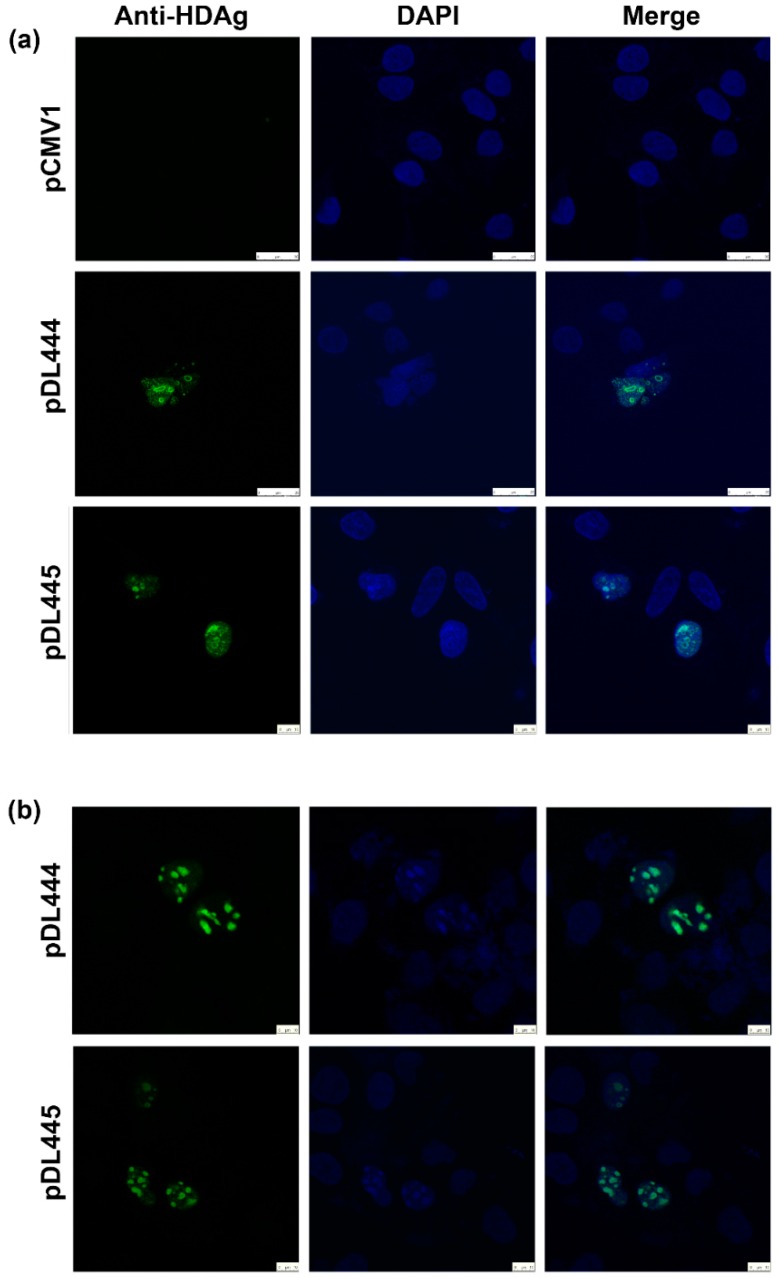
Detection of small (S) and large (L) HDV antigens in Huh7.5 cells by the immunofluorescent staining with polyclonal rabbit antibodies specific to S-HDAg. In brief, Huh7.5 cells were transfected with pDL444 encoding S-HDAg, or pDL445 encoding large HDV antigen (L-HDAg), or control pCMV1 (as depicted on the left side). Cells were grown for 24 h and fixed with methanol-acetone (**a**); or grown for 48 h and fixed with paraformaldehyde (**b**). Vertical panels left to right: staining with anti-S-HDAg rabbit serum (#100-3a) and FITC-conjugated anti-rabbit antibodies (anti-S-HDAg; green); DAPI for nucleus (DAPI, blue); overlay of anti-S-HDAg and nuclear staining (merge). The large white bar on panel (**a**) for pCMV1 and pDL444 plasmids denote 25 µm, whereas the small bar on panel (**a**) for plasmid pDL445 and on panel (**b**) denotes 10 µm.

Finally, we sought to evaluate to applicability of the sera for the detection of HDV antigens by fluorescent/confocal microscopy. Huh7.5 cells were seeded on glass coverslips and transfected with pDL444 encoding S-HDAg, or pDL445 encoding L-HDAg and let them grow for 24 to 48 h. Two protocols were used to fix the cells. Methanol-acetone protocol was applied to fix cells 24 h posttransfection, whereas cells at 48 h posttransfection were fixed by formaldehyde. In both cases, anti-S-HDAg antibodies efficiently stained both small and large HDV antigens (Figure 4). In lines with the earlier observations [21], both antigens exhibited nucleolar localization (Figure 4).

## 3. Materials and Methods 

### 3.1. Reagents

Protease inhibitor cocktail used in the protein purification was from Sigma (St. Louis, MO, USA). The empty pCMV1 vector was purchased from Invitrogen (Carlsbad, CA, USA). The primary antibodies to β-actin were from Abcam (ab3280). HRP-conjugated secondary antibodies against rabbit (sc-2004) or mouse (sc-2005) immunoglobulins were purchased from Santa-Cruz Biotechnology (Dallas, TX, USA). FITC-conjugated anti-rabbit antibodies were from Jackson ImmunoResearch Labolatories (West Grove, PA, USA). Plasmids pDL444 and pDL445 directing the expression of S-HDAg and L-HDAg in the mammalian cells were a kind gift of David Lazinski (Tufts University, Boston, MA, USA) and Severin Gudima (University of Kansas Medical Center, KS, USA). Human hepatoma Huh7.5 cell line was kindly provided by C.M. Rice (The Rockefeller University, NY, USA) and Apath L.L.C. (NY, USA).

### 3.2. Plasmid Construction

The plasmid for prokaryotic expression of S-HDAg was constructed based on the pET-21d vector (Novagen, Madison, WI, USA). The fragment encoding S-HDAg was amplified from the plasmid pDL444 [22] using primers 5′-AAAAAAAACCATGGCTCGGTCCGAGTCG-3′ and 5′-ATAAAGCTTTCAGTGGTGGTGGTGGTGGTGTGGAAATCCCTGGTTTCCC-3′, digested with *Nco*I and *Hind*III endonucleases and cloned into the respective sites of pET-21d vector. The structure of the resulting plasmid pET-21d-SHDAg was verified by sequencing using ABI PRISM^®^ BigDye™ Terminator v. 3.1 reagents with the subsequent analysis of products on an automatic Applied Biosystems 3730 DNA Analyzer (CCU “Genome”, EIMB, Moscow, Russia).

### 3.3. Protein Expression and Purification

The plasmid pET-21d-SHDAg was transformed into Rosetta (DE3) *E. coli* strain. A single colony was inoculated into 10 mL of LB medium supplemented with 150 mg/L ampicillin and 15 mg/L chloramphenicol and grown overnight at 37 °C. Five mL of the culture was added to 500 mL of fresh medium supplemented with the same antibiotics, and the cells were grown at 37 °C until the optical density reached 0.5–0.6 (measured at 550 nm). Protein synthesis was then induced by the addition of isopropyl-β-d-1-thiogalactopyranoside (IPTG) to the final concentration of 1 mM. Cells were grown for additional 4 h, collected by centrifugation (15 min, 3200× *g*), washed with 20 mL of buffer A (25 mM Tris-НСL, pH 7.6, 50 mM glucose, 10 mM EDTA), and stored at −70 °C.

The cell pellet was suspended in 25 mL of buffer B (25 mM Tris-НСl, рН 7.5, 300 mM NaCl, 1 mM 2-mercaptoethanol, 10% (*v*/*v*) glycerol, 1 mM PMSF, 0.1% (*v*/*v*) protease inhibitor cocktail) supplemented with 0.5% (*v*/*v*) triton Х-100. The suspension was lysed by sonication on ice (Bandelin Sono Plus apparatus, 7 × 45 s impulses with 2 min gaps). Cell debris was removed by centrifugation at the 16,000× *g* for 10 min, and the clarified lysate was loaded onto a 2-mL Ni-NTA His-NTA agarose (Qiagen, Dusseldorf, Germany) column. The eluate collected after loading was re-applied onto the column one to two times to increase target protein binding. The resin was washed with buffer B, then buffer B supplemented with 10, then 30, then 40 mM imidazole (20 mL each). The target protein was eluted with buffer B supplemented with 200 mM imidazole, and 0.5 mL fractions were collected. Level of the protein in each fraction was estimated using Coomassie R-250 dye staining. Fractions containing the highest amount of S-HDAg were pooled, dialyzed overnight against 200 mL of 25 mM Tris-HCl buffer (pH 7.5) supplemented with 300 mM NaCl, 1 mM 2-mercaptoethanol, 1 mM PMSF, and 10% (*v*/*v*) glycerol and put at +4 °C, to be used for immunization within 24 h after protein purification. For longer storage, recombinant S-HDAg dialyzed against the same buffer containing 50% (*v*/*v*) glycerol and frozen at −20 to −80 °C.

### 3.4. Rabbit Immunization

All animal experiments were performed in accordance with the Russian Federation law and approval for the rabbit immunizations issued by the local ethical committee for the animal experiments. Rabbits of the Moscow strain of grey Chinchilla (female, 2 month old, 1.5 to 1.8 kg) were obtained from the laboratory animal breeder “KrolInfo” (Orekhovo-Zuevo, Moscow region, Russia, available online: http://krolinfo.umi.ru). Animals were maintained at 20 to 22 °C and a relative humidity of 50% ± 10% on a 12-h light/dark cycle, fed with commercial rodent chow and herbal vitamin flour (Zoomir, Moscow, Russia) and provided with the tap water ad libitum. The treatment of animals was in accordance with the regulations outlined in the USDA Animal Welfare Act and the conditions specified in the Guide for care and use of laboratory animals [23].

Rabbits no. 99 and no. 100 were immunized with the injections of the recombinant S-HDAg. On day 1 animals were primed with 80 µg of S-HDAg in 400 µL PBS mixed (1:1 *v*/*v*) with the complete Freund Adjuvant (CFA), and on day 7, with the same dose administered with the incomplete Freund Adjuvant (IFA). Primes were given with 20 gauge needles as four widely separated subcutaneous injections along the back (4 times 20 µg in the total of 200 µL). Animals were boosted four times with one month intervals by intravenous injections of 40 µg of S-HDAg in 200 µL PBS mixed with IFA (1:1 *v*/*v*) split into two injections into the ear veins. Due to the protein instability, each booster injection was done with a freshly prepared protein preparation. Two control animals received same injections without S-HDAg. Rabbits were bled from the ear vein two weeks post each immunization. Additional bleedings were done within one month after the main bleedings 2–4 and dubbed 2b, 3b, 3c, 4b, and 4c, respectively. Sera were aliquoted and stored at −20 °C until further use.

### 3.5. ELISA

Rabbit sera were assessed for the levels of antibodies against S-HDAg. For this, freshly purified S-HDAg was diluted in PBS at 0.3 μg/mL and coated onto 96-well MaxiSorp plates (Nunc, Roskilde, Denmark) by overnight incubation at 6–8 °C. Coated plates were blocked with PBS containing 1% BSA for 1 h at room temperature. Rabbit sera were serially diluted in the range 10^3^ to 10^9^ in PBS containing 0.05% Tween 20, 0.5% BSA, 2% goat serum (all from Sigma) (Scan buffer). The prediluted rabbit sera were applied on the plates in the amount of 100 µL per well and incubated overnight at 6–8 °C. After incubation, sera were discarded, plates were washed six times with PBS containing 0.05% Tween 20, and filled 100 µL per well with the horseradish peroxidase-conjugated goat anti-rabbit secondary antibody (DAKO, Glostrup, Denmark) diluted in Scan buffer. After 1.5 h incubation at 37 °C, secondary antibodies were discarded, plates were washed six times as above and treated 100 µL per well with the liquid substrate 3,3′,5,5′-tetramethylbenzidine (TMB) pre-diluted 1:10 in the substrate buffer (both from Medico-Diagnostic Laboratory, Moscow, Russia). Color was developed for 15 min at room temperature in the dark, reaction was stopped by adding 50 µL per well of 2.5 M sulfuric acid. Plates were read on the automatic reader (Multiscan EX, Thermo Electron Corporation, Waltham, MA, USA) at a dual length of 450 versus 620 nm. The average optical absorption values demonstrated by the naïve and control rabbit sera at each of the dilutions, and standard deviation (STDEV) of each individual serum from the average were calculated. Cut-off values to discriminate sera containing the specific antibodies from the negative sera were defined as an average OD450–620 of the preimmune and control rabbit sera at the given dilution plus 3 STDEV. OD450–620 values of all serum samples and the cut-off values were plotted on a logarithmic scale to generate the titration curves. The end-point titer of specific antibodies was determined as the dilution factor at which the optical absorption of a sample crossed the cut-off curve.

### 3.6. Cell Culture and Transfection

Huh7.5 cells were maintained as previously described [24,25]. The cells were seeded onto 6-well plates at a density of 3 × 10^5^ cells/well, 24 h prior to transfection. Transfections were carried out using Turbofect reagent (Thermo Scientific, Rockford, IL, USA). The complexes were obtained by mixing 2 μg/well of plasmids with 4 μL/well Turbofect in 400 μL/well OPTI-MEM medium with subsequent incubation at room temperature for 25 min. The complexes were added to the cells, 3 h later the medium was replaced with the fresh one, and 30 or 48 h posttransfection the cells were harvested by scrapping and stored at −70 °C.

### 3.7. Western Blot Analysis

The recombinant S-HDAg was dissolved or a pellet of Huh7.5 cells was resuspended in the Laemmli buffer, incubated at 100 °C for 5 min and applied onto 15% SDS-polyacrylamide gel. After electrophoresis, the proteins were transferred to a Hybond ECL membrane (GE Healthcare). The membrane was blocked with 5% (*w*/*v*) non-fat milk in PBS supplemented with 0.05% (*v*/*v*) Tween 20 (PBST), incubated with anti-S-HDAg rabbit serum diluted 1000 to 1:60,000 in PBST at 4 °C overnight, washed with PBST (3 × 7 min), incubated with HRP-conjugated anti-rabbit antibodies (1:3000 dilution) for 1 h at room temperature and washed with PBST (3 × 7 min). The bands were visualized using Pierce ECL Western Blotting Substrate or Super Signal West Femto Maximum Sensitivity Substrate reagents from Termo Scientific using ChemiDoc MP equipment (Bio-Rad, Hercules, CA, USA).

### 3.8. Immunofluorescence

Huh7.5 were seeded at 15,000 cell/well density on the glass coverslips placed into a 6-well plate. On the next day, the cells were transfected with pDL444, or PDL445, or control pCMV1 plasmids using a Lipofectamin LTX reagent (Termo Scientific) according to the manufacturer’s instructions. Twenty four hours later, the cells were washed with PBS, fixed with a methanol:acetone (1:1) mixture, kept at −20 °C overnight, and then rehydrated in PBS by incubation during 30 min at room temperature. Alternatively, 48 h posttransfection the cells were washed, fixed by incubation with 2% formaldehyde for 15 min at room temperature, treated with 0.5% Triton X-100 for 5 min, and washed with PBS. Following fixation, the cells were stained for 1 h at room temperature with hyperimmune rabbit serum #100-3a (Figure 2) diluted in the Blocking buffer (PBS, 2% (*w*/*v*) bovine serum albumin, 0.2% (*v*/*v*) Tween 20, 10% (*v*/*v*) glycerol). After that, cells were washed with PBS three times and incubated for 45 min with goat anti-rabbit antibodies conjugated with FITC diluted in the Blocking buffer. After washing with PBS, the cells were incubated with 300 nM solution of DAPI in PBS for 5 min and rinsed with PBS. Finally, coverslips were mounted on the microscopic slides with cells facing the slide, using VECTASHIELD^®^ Antifade Mounting Medium (Vector labs, Burlingame, CA, USA). The fluorescence was analyzed on the confocal laser scanning microscope Leica TCS5 with a 63× objective.

## 4. Conclusions

To conclude, we have expressed and purified small antigen of hepatitis delta virus S-HDAg that was further used to obtain specific anti-S-HDAg antibodies in an extremely high titer. Antibodies were successfully applied for the sensitive detection of both small and large HDV antigens in ELISA, Western blotting and immunofluorescence assays. These antibodies can be further used for medical studies as well as for the HDV-focused research.

## Supplementary Materials

Supplementary materials can be found at www.mdpi.com/1422-0067/17/10/1721/s1.
