# Supplementary material for: Prokaryotic Expression, Purification and Immunogenicity in Rabbits of the Small Antigen of Hepatitis Delta Virus
Source: Int J Mol Sci. 2016 Oct 20;17(10):1721. doi: 10.3390/ijms17101721 (PMC5085752; doi:10.3390/ijms17101721)
Supplement: Supplementary file 1 [file ijms-17-01721-s001.pdf]

Supplementary Materials: Prokaryotic Expression, Purification and Immunogenicity in Rabbits of Small Antigen of Hepatitis Delta Virus

Vera L. Tunitskaya, Olesja V. Eliseeva, Vladimir T. Valuev-Elliston, Daria A. Tyurina, Natalia F. Zakirova, Olga A. Khomich, Martins Kalis, Oleg E. Latyshev, Elizaveta S. Starodubova, Olga N. Ivanova, Sergey N. Kochetkov, Maria G. Isaguliants and Alexander V. Ivanov

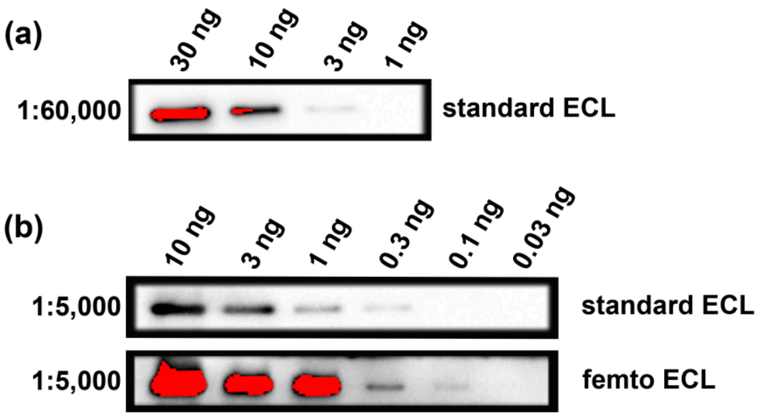

Figure S1. Detection of HDV antigens using Western blotting with polyclonal rabbit sera specific to S-HDAg. Detection of the recombinant S-HDAg in the amounts 30 to 0.3 ng using rabbit serum #99-3b diluted as 1:60,000 (**a**) or 1:5000 (**b**) by the standard ECL or sensitive femto ECL reagents (S-HDAg amounts are shown above the lanes in panels **a**, **b**).

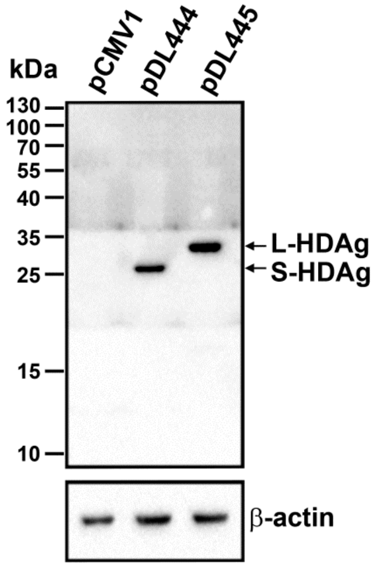

Figure S2. Expression of small and large HDV antigens in Huh7.5 cells. Huh7.5 cells were transfected with pDL444 or pDL445, respectively, and control cells, with empty vector pCMV1 and grown for additional 48 h. Blots were first stained with anti-S-HDAg serum diluted at 1:5000 (**upper** panel), then stripped and re-stained with mouse monoclonal against actin (**lower** panel).
